# Facteurs de risque de l'inobservance thérapeutique chez les patients schizophrènes: étude cas- témoins'

**DOI:** 10.11604/pamj.2015.20.273.3380

**Published:** 2015-03-20

**Authors:** Chadya Aarab, Fatima Elghazouani, Rachid Aalouane, Ismail Rammouz

**Affiliations:** 1 Centre psychiatrique universitaire Ibn Alhassan, CHU Hassan II Fès, Maroc; 2 Laboratoire Neuroscience, Faculté de Médecine et de Pharmacie, Université Sidi Mohamed Ben abdellah, Fès, Maroc; 3 Centre Psychiatrique Universitaire, Faculté de Médecine et de Pharmacie Oujda, Maroc

**Keywords:** Observance thérapeutique, schizophrénie, facteurs de risque, treatment adherence, schizophrenia, risk factors

## Abstract

Les progrès réalisés dans le traitement de la schizophrénie n'ont jusqu'ici pas modifié de manière radicale l'importance de l'adhésion des patients à leur médication. L'objectif de ce travail est d'identifier les facteurs de risque de l'abandon du traitement sur un échantillon marocain de malades schizophrènes. C'est une étude transversale menée au centre psychiatrique universitaire de Fès sur une période de 4 mois. Les malades inclus présentaient une schizophrénie ou un trouble schizo-affectif, sélectionnés en deux groupes observant et non observant. L’évaluation de l'observance a été faite par un hétéro-questionnaire comprenant une liste de causes d'abandon du traitement avec des réponses par oui ou non et à l'aide de l’échelle MARS (Medication Adherence Rating Scale). Le traitement statistique des résultats a été réalisé par le logiciel Epi Info version 3.5.1. On a recruté 164 participants, 112 étaient des malades non observants à leur traitement (cas) et 52 patients bien observants (témoins). L’âge moyen est 31 ans, avec une prédominance masculine. Les facteurs de risque d'inobservance thérapeutique sont: l’âge jeune, le sexe masculin, le célibat, les troubles addictifs. Les principales raisons d'abandon du traitement sont le changement fréquent du médecin, le manque d'informations sur la maladie, un mauvais insight et les effets secondaires des antipsychotiques. Le groupe de schizophrènes non adhérents à leur traitement pharmacologique avait un score élevé à l’échelle MARS dans 80% cas. Ces résultats doivent être pris en considération par le personnel soignant pour optimiser l'observance thérapeutique chez les patients souffrant de schizophrénie.

## Introduction

L'observance, c'est la concordance entre le comportement d'un patient et les prescriptions médicales - médicamenteuses ou non - qui lui ont été recommandées. Ce phénomène, difficile à évaluer, a cependant des conséquences médicales et économiques considérables. Le thème de l'observance thérapeutique a acquis lors de ces dernières années une place de plus en plus importante dans le champ des travaux sur la schizophrénie. Ainsi la littérature psychiatrique est assez riche en ce thème avec ses différents aspects: prévalence, facteurs de risque, évaluation des outils de mesure et interventions cognitives, comportementales ou familiales. Les progrès réalisés dans le traitement de la schizophrénie n'ont jusqu'ici pas modifié de manière radicale l'importance de l'adhésion des patients à leur médication. La plupart des patients souffrant de schizophrénie présentent une observance médicamenteuse partielle ou nulle [1, 2]. Seul un tiers d'entre eux suit leur traitement tel qu'il a été prescrit [3]. La non observance au traitement a des conséquences cliniques certes, mais aussi socioéconomiques séjours hospitaliers prolongés, utilisation excessive de soins de santé [4–6]. Des études ont abordé le coût d'inobservance pour la société; une revue de littérature a déchiffré 70 études faites entre 1995 et 2007 et avec le coût dû aux hospitalisations a augmenté de 1392 millions de dollars en 1995 à 1826 millions en 2005 [7]. Les travaux dans les pays du sud sont relativement rares, et on ne dispose pas assez de données propres à la prévalence et les facteurs prédictifs surtout d'ordre socio-culturel et environnemental. Au Maroc, la problématique de l'observance thérapeutique des malades schizophrènes est clairement perçue à travers les nombreuses réhospitalisations et rechutes des malades. Il nous a semblé intéressant d’étudier et d'explorer ce problème à travers une étude menée au service psychiatrique universitaire de Fès au Maroc à fin de dépister les causes les plus fréquentes de l'abandon du traitement pharmacologique chez les patients schizophrènes, mettre en évidence des facteurs de risques sociodémographiques, cliniques et thérapeutiques de la mauvaise observance et avoir une réflexion autour des solutions envisageables pour traiter cette non -compliance.

## Méthodes

C'est une étude transversale réalisée au service de psychiatrie du centre hospitalier universitaire de Fès (Maroc) sur une période de quatre mois. Critères d'inclusion: âge >18 ans, les malades ayant rempli les critères de DSM IV de schizophrénie et trouble schizo-affectif, ayant pris un traitement neuroleptique au moins une année, toutes les formes cliniques sont inclues. Les participants sont sélectionnés en deux groupes observant et non observant. Pour le groupe des malades observants: observance thérapeutique durant la dernière année jugée par le médecin traitant en se basant sur la régularité de renouvellement d'ordonnances, par le patient lui-même et par sa famille.

Pour les critères des malades non observants: patients hospitalisés pour rechute survenant après un arrêt d'au moins un mois du traitement. Les critères d'exclusion: malade non coopérant ou refusant l'entretien, comorbidités neurologiques ou retard mental, présence de troubles cognitifs sévères. Le recueil des données a été fait par une fiche d'exploitation comprenant les données sociodémographiques, les antécédents médico-chirurgicaux et psychiatriques, caractéristiques cliniques de la maladie, le nombre des hospitalisations antérieures et l'usage de substances. L’évaluation de l'observance a été d'abord faite par un hétéro-questionnaire comprenant une liste de causes d'abandon du traitement avec des réponses par oui ou non. L'examinateur pose des questions fermées avec clarté en langue dialectique. La Durée de la passation est de 30mn. Le deuxième outil de mesure est un Auto-questionnaire: MARS (Medication Adherence Rating Scale). Il s'agit d'une variable quantitative discrète qui comporte 10 items auxquels les patients répondent par oui ou non, elle présente une bonne validité et fiabilité. Les questions se rapportent au comportement du patient concernant son traitement d'une part et aux perceptions subjectives par rapport au traitement d'autre part. Le traitement statistique des résultats a été réalisé par le logiciel Epi Info version 3.5.1 Aspect éthique: la confidentialité des informations a été respectée en utilisant un questionnaire anonyme. La mise en route de l’étude a été accordée par le comité d’éthique de la faculté de médecine de Fès.

## Résultats

On a recruté 164 malades, l’âge moyen est de 31 + /- 1,41ans. 112 patients dans le groupe non observant (cas) et 52 malades dans le groupe observant (témoins). On a noté une prédominance masculine. Les patients non observants sont moins jeunes que les observants. Ainsi que le pourcentage des malades vivant seuls est très élevé dans la population des non observants. Les autres différences soci-odémographiques entre les deux groupes sont illustrées dans le Tableau 1. Concernant les données cliniques, le groupe des témoins a significativement moins d'antécédents judicaires (2% vs 31,25%) et addictifs (15,4% vs 20,54%) par rapport au groupe de schizophrènes inobservants. On a noté aussi une prédominance significative de la forme paranoïde dans le groupe des patients inobservants par rapport au groupe témoin (70,5% vs 9,6%). En revanche, on n'a pas constaté de différences significatives entre les deux groupes concernant l’âge de début de la schizophrénie, la présence d'antécédents personnels de tentative de suicide ou familiaux de psychoses. Pour le groupe des non-observants, les principales raisons d'abandon du traitement sont le changement fréquent du médecin, le manque d'informations sur la maladie et son traitement, un insight pauvre par rapport à la pathologie et enfin les effets secondaires des antipsychotiques. Alors que les autres étiologies sont moins représentées dans notre travail (Tableau 2). A propos de l’échelle de MARS le groupe de schizophrènes non adhérents à leur traitement pharmacologique avait un score élevé de MARS dans 80% cas, alors que le groupe de témoins avait un score bas (Tableau 3).

**Tableau 1:** Données sociodémographiques des 2 groupes

Données sociodémographiques	CAS N = 112	TEMOINS N = 52	P
Moyen âge	32.18 ± 8,7 ans	37.8 ± 9,4 ans	0.0004
Sexe masculin	84,8%	69,2%	0,0013
Sexe féminin	15,2%	30,8%	
Célibataire	71%	40,4%	
Marié	18%	28.8%	0.0020
Divorcé/veuf	4%	11.5%	
Jamais scolarisé	37%	25%	NS
Primaire	36%	46,2%	
Secondaire	23%	19,2%	
Universitaire	4%	9.6%	
Vit avec la famille	70%	96%	0,0000
Vit seul	30%	4%	
Urbain	61%	82.7%	0.003
Rural	39%	17, 3%	
Revenu mensuel			
< 3000 DH	69.6%	50%	
3000-5000 DH	22,3%	44,2%	0.0163
>5000 DH	8%	5,8%	

NS: non significatif

**Tableau 2:** Les étiologies de non observance au traitement par ordre décroissant

Causes de l'abandon du traitement	%
Vous changez fréquemment de médecin?	89.28%
Vous manquez d'informations sur votre maladie et sur les médicaments prescrits?	79.46%
Vous ne vous considérez pas malade?	65.17%
Vous trouvez que les médicaments ont des effets de somnolence, de fatigue, et par conséquent vous ne pouvez pas travailler ou poursuivre vos études?	61.60%
vous trouvez que les médicaments sont responsables des troubles neuro végétatifs tels: vision flou, prise de poids, vertige, sécheresse buccale, …?	60.71%
Vous interrompez votre traitement à cause des effets extrapyramidaux (tremblement, torticolis, rigidité…)?	59.82%
vous n'avez pas senti l'effet positif et immédiat du traitement?	59.82%
vous n'avez pas senti les effets bénéfiques des médicaments?	58.03%
vous considérez la prise à long terme du traitement très fatigante et très pénible?	54.46%
Vous vous estimez guéri et n'avoir plus besoin des médicaments?	53.57%
Vous trouvez votre médecin incompétent et vous n'avez pas confiance en ses prescriptions?	51.78%
vous consommez une substance toxique qui vous aide mieux que les médicaments (cannabis, alcool, solvants …)?	50%
vous trouvez les médicaments trop chers?	50%
vous croyez que la psychothérapie est suffisante?	45.53%
vous trouvez le traitement trop lourd: multiples produits, plusieurs prises?	45.53%
Vous trouvez que votre médecin n'est pas bon et vous ne sentez pas à l'aise avec lui?	44.64%
vous trouvez que les médicaments ont des effets sur votre sexualité.	32.14%
Vous vous préoccupez de ce que pense les autres si vous prenez des médicaments prescrits par un psychiatre.	28.57%
vous pensez que la vie ne vaut pas la peine d’être vécue, alors à quoi servirait votre effort de sortir de votre maladie?	25%
Votre famille vous surveille trop et vous oblige à prendre le traitement.	10.71%
Autres raisons?	2.67%

**Tableau 3:** Tableau comparatif de différents scores de MARS chez les 2 groupes

Questionnaire	Cas		Témoins	P
Vous est-t-il parfois arrivé d'oublier de prendre vos médicaments?	48,21%		43,9%	0,0530
Négligez-vous- parfois l'heure de prise d'un de vos médicaments?	77,68%		7,7%	0,0000
Lorsque vous vous sentez mieux, interrompez-vous parfois votre traitement?	81,25%		5,8%	0,0000
Vous est-t-il arrivé d'arrêter le traitement parce que vous vous sentiez moins bien en le prenant?	74,10%		3,8%	0,0000
Je ne prends les médicaments que lorsque je me sens malade	69,64%		3,8%	0,0000
Ce n'est pas naturel pour mon corps et pour mon esprit d’être équilibré par des médicaments	41,96%		5,8%	0,0000
Mes idées sont plus claires avec les médicaments	45,53%		92,3%	9-10
En continuant à prendre les médicaments, je peux éviter de tomber à nouveau malade	40,17%		88,8%	4-6
Avec les médicaments, je me sens bizarre comme un « zombie »	80,36%		15,4%	0,0000
Les médicaments me rendent lourd et fatigué	78,57%		0%	0,0000

## Discussion

Le thème de l'observance est loin d’être simple. Il implique plusieurs facteurs cliniques, sociodémographiques, culturels et autres. La considération des malades schizophrènes par une vision dichotomique en malades observants et non observants s'avère très relatif. Une étude a bien montré qu'il existe cinq prototypes de patients: les bons observants pour des raisons justes, ou des raisons fausses, les observants avec réticence, les observants passifs et les non observants « consultants » [8]. L'observance n'est pas un phénomène en tout ou rien, il existe une graduation entre une observance totale, des oublis dans les prises médicamenteuses, des erreurs de posologie ou d'horaire de prise du traitement, un refus partiel et enfin un refus complet du traitement. L'observance peut être nulle, partielle ou totale. C'est un processus dynamique qui peut évoluer vers une amélioration ou au contraire vers une dégradation au cours du temps en fonction de divers facteurs qui l'influencent [9].

La mesure de l'observance thérapeutique pose énormément de problèmes. C'est un concept difficile à évaluer et à quantifier, L’étude de Chadzynsk a bien montré qu'il existe une nette différence du taux d'observance rapporté par les psychiatres, les familles et les patients [10]. Plusieurs outils de mesure sont utilisés pour évaluer l'observance, soit par des méthodes subjectives (patient lui-même, les soignants..) ou moyens objectifs (comptage de nombre de comprimés (pilulier), dossier de pharmacie, monitoring éléctronique, ou concentrations plasmatiques). Cependant tous ces outils présentent des limites et donnent une idée approximative de l'adhésion aux traitements [11–13]. Dans notre étude, nous avons utilisé l’échelle de MARS (medication adhérence rating scale), vue sa simplicité, sa facilité d'utilisation, composée de 10 items auxquels le sujet répond par oui/non et aussi vu qu'elle s'est intéressée à la composante comportementale liée au phénomène d'observance. L'identification des facteurs prédictifs de l'observance thérapeutique demeure un problème central dans la prise en charge de la schizophrénie. Il peut s'agir de facteurs liés au malade lui-même, à la maladie ou au traitement médicamenteux. Dans notre étude le support familial se distingue comme étant un élément hautement significatif de la bonne observance, et ces résultats ont été reproduits dans d'autres études [14–16]. D'autres variables socio-démographiques tel que l’âge jeune, le sexe masculin, constituent des facteurs de risque avec un P significatif (p < 0,005) dans notre échantillon, par contre, le niveau scolaire et économique bas étaient des facteurs indépendants. L’étude de Gilmer et coll a montré que l'adhésion au traitement augmente significativement avec l’âge passant de 25% chez ceux âgés de <25 à 37% chez ceux de 25 à 59ans et 43% chez les sujets de 60 ans et plus [17]. Une autre étude sur 867 schizophrènes a comparé les caractéristiques sociodémographiques des groupe adhérent (372) et groupe non adhérent (504) aux traitements, a noté que le seul point de différence était le niveau scolaire bas chez les malades non observants [18].

L'usage de substance comme facteur d'inobservance a été mis en cause dans plusieurs études et particulièrement pour le cannabis [19]. Notre étude a révélé une proportion importante d'usage de cannabis chez les non observants (P = 0,000), ce qui constitue un véritable handicap dans la prise en charge de la maladie. Les sujets schizophrènes qui présentent une comorbidité addictive forment un groupe à haut risque de non-observance. Buckley et al. ont expliqué que les toxiques entraînent des altérations des systèmes dopaminergiques au niveau mésolimbique, ce qui rendait les patients résistants à l'action des neuroleptiques et grèverait le pronostic [20]. Plusieurs auteurs ont démontré que l'insight est la principale variable associée significativement au niveau d'observance et la perception subjective du traitement indépendamment des autres caractéristiques démographiques et cliniques [21, 22]. Dans notre travail, un mauvais insight était largement rapporté par les malades non observants (65,17%). En effet, l'absence de prise de conscience de la maladie « insight » va de pair avec la difficulté liée à la perception d'un bénéfice thérapeutique. D'autres paramètres cliniques ont été évalués dans notre étude en particulier l’âge de début de la maladie, antécédents de tentatives de suicide ou antécédents familiaux de troubles psychotiques mais sans différence significative entre les deux groupes. Par ailleurs, concernant les formes cliniques de la schizophrénie, on a noté une prédominance de la forme paranoïde dans le groupe non observant, alors que le trouble schizoaffectif est largement présenté dans le groupe de malades observants. Les résultats de la littérature sont contradictoires dans ce sens, Les travaux de Van Putten et al ont montré qu'il n'existe aucun lien net entre sous-type paranoïde et qualité de la compliance. Les paranoïdes persécutés ou sous influence sont compliants dans 85% des cas, tandis que 92% des paranoïdes mégalomanes refusent tout traitement [23]. Un autre facteur bien impliqué dans l'observance est la tolérance des médicaments, les effets secondaires des antipsychotiues représentent ainsi une cause majeure d'arrêt de traitement en particulier de nature neurologique ou encocrinométabolique. Ces données sont rapportées dans notre étude et dans de nombreux travaux [18]. L'alliance thérapeutique est parmi les facteurs majeurs impliqués dans l'adhésion aux soins. L'obtention d'une bonne observance passe par l'obtention d'une bonne relation thérapeutique; cette dernière est directement liée à l’écoute et à l'intérêt accordés au malade, et pas seulement à ses symptômes [24]; dans notre travail 44% des patients interrogés non observants ont rapporté qu'ils se sentaient mal à l'aise avec leur médecin et 89% changent fréquemment de médecins.

Certains facteurs d'inobservance sont peu évoqués dans la littérature et par conséquent rarement pris en considération dans la pratique quotidienne; le changement d'une molécule originale par un médicament générique pèse parfois lourd sur l'observance [25, 26]. Il existe aussi des raisons socioculturelles et religieuses qui interférent avec la perception du malade vis-à-vis des médicaments [17, 27]. Le facteur social et économique s'impose aussi comme l'un des éléments prédictifs de l'observance. Les difficultés financières, des barrières dans l'accès aux soins, la désinsertion sociale accroissent la non-compliance [28]. Ainsi dans notre contexte marocain le manque importants en personnels et structures psychiatriques aggravent encore ce problème, en effet, une population de 33 millions d'habitants ne dispose que d'environ 320 médecins psychiatres dont 124 installés dans le secteur privé, 740 infirmiers spécialisés en psychiatrie et 80 psychologues cliniciens, et une capacité litière très limitée [29]. La compréhension de l'inobservance nécessite une approche multifactorielle, intégrant à la fois des facteurs biomédicaux, psychosociaux et environnementaux. La prise en compte de ces déterminants doit permettre la mise en place d'interventions visant à améliorer l'adhésion thérapeutique [30].

Ainsi plusieurs stratégies ont été proposées pour améliorer l'adhésion aux traitements. Des consensus ont été élaborés par des experts ont noté l´importance de distinguer les patients qui refusent de prendre les médicaments de ceux qui acceptent, mais ne sont pas en mesure de prendre leur médicament comme prescrit par oubli, mauvaise compréhension des instructions, ou des problèmes financiers ou environnementaux, car cela va affecter le type d'intervention envisagée. Les experts ont également recommandé de parler avec la famille ou le personnel soignant, si le patient donne l´autorisation, ainsi que l´utilisation de mesures plus objectives (par exemple, le décompte de pilules, les dossiers de la pharmacie, des contenants de pilules intelligentes, ou les concentrations plasmatiques des médicaments). L´utilisation d´une échelle d´auto-évaluation validée peut également aider à améliorer la précision de l'observance. Pour les patients qui apparaissent adhérents à la médication, les experts ont recommandé des évaluations mensuelles pour l´adhésion, des évaluations supplémentaires s'il ya un changement notable symptomatique [31, 32]. La simplicité du traitement médicamenteux par la réduction du nombre de prises et la prescription des doses optimales des antipsychotiques a aussi son effet positif sur l'observance [33, 34]. L'intérêt des antipsychotiques atypiques et les neuroleptiques à action prolongée a été clairement démontré dans plusieurs études [35–40]. Les programmes psychoéducatifs visant à améliore l'observance et à diminuer la fréquence des réhospitalisations, permettent une compensation relative des déficiences liées aux troubles psychiatriques par une implication du patient dans son propre parcours thérapeutique, mais aussi celle de son entourage. Cette démarche qui induit une forte adhésion thérapeutique du patient est mise en place selon ses besoins et son profil [41]. Il reste encore parmi les actions bénéfiques sur l'observance, les visites à domiciles des malades, Kampman et al. ont montré qu'une prise en charge sectorielle renforcée de type visites à domiciles par une infirmière toutes les deux à quatre semaines diminuait le nombre d'hospitalisation et améliorait l'observance [42]. Enfin le facteur clé est d'améliorer l'alliance thérapeutique qui permet d'identifier les problèmes potentiels d'adhésion [43]. L´application de ces principes et stratégies devrait contribuer à l'amélioration générale des soins des patients atteints de schizophrénie.

### Limites du travail

Notre méthode de déterminer les deux groupes observant et non observant s'est basée essentiellement sur l'arrêt ou non du traitement objectivé par le médecin, le patient et la famille. Alors que l’échelle de MARS n'a été qu'ultérieurement utilisée comme moyen comparatif entre les deux groupes. Le recrutement des patients observant est difficile dans une unité hospitalière ce qui a aboutit à une inégalité des deux groupes et par conséquent, il a limité notre étude. Beaucoup de paramètres considérés comme influençant l'observance médicamenteuse, n'ont pas été pris en compte dans notre étude notamment les modalités thérapeutiques, la présence ou non de symptômes dépressifs et le dysfonctionnement familial. Tout de même c'est un étude comparative cas-témoins de malades schizophrènes qui nous a permis de mettre en évidence des facteurs de risque liés à la mauvaise observance thérapeutique dans notre contexte marocain afin d'envisager des interventions spécifiques et plus adaptées à nos conditions.

## Conclusion

L'observance thérapeutique est un objectif essentiel de la prise en charge de la schizophrénie. Son évaluation demeure plus compliquée que sa réalisation. En effet, le repérage des facteurs de mauvaise observance optimise la prise en charge thérapeutique en adoptant des stratégies thérapeutiques propres à chaque patient qui doit à son tour participer à son projet thérapeutique.
